# Lipopolysaccharide Regulates Pro- and Anti-Inflammatory Cytokines, Corticosterone, and Melatonin in Toads

**DOI:** 10.1093/iob/obab025

**Published:** 2021-08-28

**Authors:** L F Ferreira, P G Garcia Neto, S C M Titon, B Titon, S M Muxel, F R Gomes, V R Assis

**Affiliations:** Faculdade de Filosofia, Ciências e Letras do Centro Universitário Fundação Santo André, Avenida Príncipe de Gales, 821, Santo André, SP 09060-650, Brasil; Departamento de Fisiologia, Instituto de Biociências, Universidade de São Paulo, Rua do Matão, trav. 14, 101, São Paulo, SP 05508-090, Brasil; Departamento de Fisiologia, Instituto de Biociências, Universidade de São Paulo, Rua do Matão, trav. 14, 101, São Paulo, SP 05508-090, Brasil; Departamento de Fisiologia, Instituto de Biociências, Universidade de São Paulo, Rua do Matão, trav. 14, 101, São Paulo, SP 05508-090, Brasil; Departamento de Fisiologia, Instituto de Biociências, Universidade de São Paulo, Rua do Matão, trav. 14, 101, São Paulo, SP 05508-090, Brasil; Departamento de Fisiologia, Instituto de Biociências, Universidade de São Paulo, Rua do Matão, trav. 14, 101, São Paulo, SP 05508-090, Brasil; Departamento de Fisiologia, Instituto de Biociências, Universidade de São Paulo, Rua do Matão, trav. 14, 101, São Paulo, SP 05508-090, Brasil; Departamento de Fisiologia, Instituto de Biociências, Universidade de São Paulo, Rua do Matão, trav. 14, 101, São Paulo, SP 05508-090, Brasil

## Abstract

Glucocorticoids and melatonin (MEL) show integrated and complex immunomodulatory effects, mostly described for endotherms, yet underexplored in amphibians. In this context, the RT-qPCR of molecules mediating inflammatory processes in amphibians is a valuable tool to explore the relationships among molecular biology, endocrine mediators, and immune response in these animals. In this study, toads (*Rhinella diptycha*) received an intraperitoneal saline injection or lipopolysaccharide (LPS; 2 mg/kg). Six hours post-injection, we analyzed plasma corticosterone (CORT) and MEL levels and pro- and anti-inflammatory molecules (IL-1β, IL-6, IL-10, IFN-γ, and C1s). We found increased CORT and decreased MEL levels in response to LPS. Also, IL-1β, IL-6, and IL-10 were upregulated in LPS-injected toads compared with saline-injected toads. Overall, our results demonstrate an LPS-induced inflammatory response with endocrine and immune modulation in *R. diptycha* toads, exhibiting expected patterns for an inflammatory stimulus within this time frame (6 h post-injection). Toads were responsive to LPS by secreting different cytokines, such as proinflammatory cytokines IL-1β and IL-6, related to immune cell attraction to inflammatory sites and the anti-inflammatory cytokine IL-10, which limits the rate of leukocyte infiltration, inflammation, and downregulates the expression of proinflammatory cytokines. Increased circulating CORT levels are probably associated with the activation of the hypothalamus-pituitary-interrenal axis by the LPS and the endocrine actions of IL-6. Furthermore, decreased circulating MEL levels are likely due to inhibited MEL secretion by the pineal gland by inflammatory stimuli, indicating the activation/existence of the immune-pineal axis in amphibians.

## Resumo em português

Os glicocorticoides e a melatonina (MEL) apresentam efeitos imunomoduladores integrados e complexos, principalmente descritos para endotérmicos, porém pouco explorados em anfíbios. Nesse contexto, o RT-qPCR de moléculas mediadoras de processos inflamatórios em anfíbios é uma ferramenta valiosa para explorar as relações entre biologia molecular, mediadores endócrinos e resposta imune nesses animais. Neste estudo, sapos (*Rhinella diptycha*) receberam uma injeção intraperitoneal de solução salina ou lipopolissacarídeo (LPS; 2 mg/kg). Seis horas após a injeção, analisamos as concentrações plasmáticas de corticosterona (CORT) e MEL e moléculas pró e anti-inflamatórias (IL-1β, IL-6, IL-10, IFN-γ e C1s). Encontramos aumento de CORT e diminuição da concentração de MEL em resposta ao LPS. Além disso, IL-1β, IL-6 e IL-10 foram reguladas positivamente em sapos injetados com LPS em comparação com os sapos injetados com solução salina. No geral, nossos resultados demonstram uma resposta inflamatória induzida por LPS com modulação endócrina e imunológica em sapos *R. diptycha*, exibindo padrões esperados para um estímulo inflamatório dentro deste período (6 h pós-injeção). Os sapos foram responsivos ao LPS secretando diferentes citocinas, como as citocinas pró-inflamatórias IL-1β e IL-6, relacionadas à atração de células imunes para os sítios inflamatórios e a citocina anti-inflamatória IL-10, que limita a taxa de infiltração leucocitária, inflamação, e diminui a expressão de citocinas pró-inflamatórias. As concentrações elevadas de CORT circulante estão provavelmente associados à ativação do eixo hipotálamo-hipófise-interrenal pelo LPS e às ações endócrinas da IL-6. Além disso, a diminuição da concentração circulante de MEL é provavelmente devida à inibição da secreção de MEL pela glândula pineal por estímulos inflamatórios, indicando a ativação/existência do eixo imune-pineal em anfíbios.

## Introduction

Innate immunity is the first line of defense against pathogens, including humoral (e.g., acute phase proteins, antimicrobial peptides, complement components, lysozymes, natural antibodies) and cellular components (e.g., macrophages, neutrophils, thrombocytes, dendritic cells, natural killer cells), which have the function of eliminating pathogens and infected cells by phagocytosis or direct cytotoxicity (Abbas et al. 2014; Murphy and Weaver 2017). Most organisms rely on innate immune mechanisms for survival, including inflammation (Dinarello 2018). Different stimuli can elicit an inflammatory response (e.g., physical injury, tissue damage, pathogenic infection), activating several proinflammatory mediators to remove the damaging agent and restore tissue structure and function (Beck et al. 2009). Immune cells involved in the inflammatory process can sense the surrounding environment, respond to various proinflammatory stimuli, such as pathogen/danger-associated molecular patterns (PAMPs/DAMPs), and initiate cytokine and chemokine cascades (Standiford et al. 1990; Beck et al. 2009). The complement system is an essential part of the innate and adaptive immune system, eliminating pathogens, promoting inflammation, and eliminating necrotic and apoptotic cells (Nakao et al. 2011). The complement component C1s is a serine protease and plays a crucial role in innate immunity via activation of the classical complement cascade system (Godahewa et al. 2015). Increased inflammatory mediators (e.g., tumor necrosis factor-alpha [TNF-α], interleukin [IL]-1β, and IL-6) are essential for inflammation progression, although returning to homeostasis is crucial to avoid the onset of unfavorable chronic inflammation (Pascual and Glass 2006; Beck et al. 2009). To control the course of the inflammatory process, modulatory and anti-inflammatory cytokines such as IL-10 and IL-4 are released (Beck et al. 2009). The inflammatory response can be divided into three sequential phases: (1) the alarm phase: release of inflammatory mediators; (2) the mobilization phase: leukocyte infiltration to the injured site; and (3) the resolution phase: tissue clearance of cellular debris (Cain and Cidlowski 2017). PAMPs and DAMPs induce inflammatory mediators, including cytokines, during the alarm phase. During the mobilization phase, inflammatory mediators induce the adhesion molecules on the endothelium to recruit leukocytes toward the inflammatory sites. Finally, during the resolution phase, macrophages clear debris and secrete anti-inflammatory factors, promoting wound healing (Cain and Cidlowski 2017).

The immune response is also modulated by different sources of physiological mediators, including hormones (Webster et al. 2002). Two of the most studied endocrine mediators of the immune system are glucocorticoids and melatonin (MEL). Glucocorticoids are steroid hormones with pleiotropic effects, as they modulate several physiological processes, including metabolism, development, and inflammation (Cain and Cidlowski 2017). The activation of the hypothalamus-pituitary-adrenal/interrenal (HPA/I) axis modulates the immune/inflammatory response (Sapolsky et al. 2000; Dhabhar 2014), given that virtually all the components of the immune response have glucocorticoids receptors (Chrousos 1995; Charmandari et al. 2005). In mammals and birds, the general metabolic effects of chronic stimulation of the HPA/I axis ​promote individual fitness reduction, making them more susceptible to diseases. Increases in glucocorticoids reduce lymphocytes number, decreasing antibody levels, thereby lowering viruses resistance (Siegel 1980; Reeder and Kramer 2005). On the other hand, similar increases in glucocorticoids seem to increase the resistance to bacterial infections, which is mediated by macrophages and neutrophils/heterophils. These apparent contradictions are due to the effects of glucocorticoids on the particular pathogen or the immune defense mechanisms. In diseases where the major pathology involves local or general inflammation or endotoxin formation, glucocorticoids often appear to be beneficial despite lowered antibody levels (Siegel 1980; Reeder and Kramer 2005).

Even though less frequently explored, the interactions between the HPA/I axis in non-mammalian animals have been studied. Pijanowski et al. (2015) found that the differences in sensitivity to stress influence the immune response in four different carp (*Cyprinus carpio L*.) lines. Fishes more susceptible to parasites also exhibited the highest stress response, consequently affecting survival, complying with findings in mammals. In a recent paper, Titon et al. (2021b) showed that in adult toads (*Rhinella icterica*), the innate humoral response (bacterial killing ability) was not affected by short-term or long-term stressors, neither exogenous glucocorticoid application. In contrast, one innate cellular response (skin edema) increased, while another (phagocytosis) increased or decreased depending on the stressor applied (Titon et al. 2021b). In addition, Billig et al. (2020) reported that southern leopard frog (*Lithobates sphenocephalus*) tadpoles treated with glucocorticoid had greater plasma bacterial killing ability than controls after a short-term and long-term exposure to exogenous glucocorticoid. On the other hand, tadpoles treated with the hormone had lower IgM and IgY after 12 weeks (Billig et al. 2020). Thus, both studies show that the increase in glucocorticoid has differential effects on anuran's immunity.

The major action of chronically elevated glucocorticoids is to switch off multiple activated inflammatory genes that encode for cytokines, chemokines, adhesion molecules, inflammatory enzymes, and receptors (Barnes and Adcock 2003; Barnes 2010) through inhibition of transcription factors, such as activator protein-1 (AP-1) and nuclear factor-κB (NF-κB) (Barnes and Adcock 2003; Beck et al. 2009). In contrast, glucocorticoids stimulate the production of anti-inflammatory cytokines, such as IL-10 and IL-4 (Elenkov and Chrousos 2002), to resolve the inflammatory process, restore homeostasis, and prevent an overreaction (Cruz-Topete and Cidlowski 2015). Moreover, acute exposure to glucocorticoids enhances the peripheral immune response (Dhabhar 2002) and induces the expression of toll-like receptor 2 (TLR2) (Busillo and Cidlowski 2013). Together, the abovementioned and additional literature (Silverman et al. 2005; Martin 2009; Dhabhar 2014) demonstrate the complexity of the glucocorticoid/stress-mediated immunomodulation, showing enhancing/protective effects of acute/short-term stress and the suppressive/deregulatory effects over chronic/long-term stress exposure.

Melatonin (MEL) is a hormone derived from the amino acid tryptophan, which is produced by the pineal gland and is responsible for synchronizing many physiological processes, including the immune response (Kepka et al. 2015). The neuroendocrine system has daily rhythmicity (Buijs et al. 2003); and glucocorticoids and MEL play notable roles in the sleep–wake cycle in vertebrates (Tan et al. 2010; Dumbell et al. 2016). Furthermore, it was recently discovered that MEL is produced by many other cells and organs, including immune cells (reviewed in Markus et al. 2018). The MEL production from the pineal gland and extrapineal sites may lie on the transcription factor, NF-κB, and its binding to κB responsive elements in the Aanat (aralkylamine *N*-acetyltransferase) promoter and first intron, blocking noradrenaline-induced MEL synthesis in pinealocytes (homodimer p50/p50) and inducing MEL synthesis in activated macrophages (c-Rel/RelA dimers) (Markus et al. 2018). Melatonin has a pivotal role in immune surveillance and the assemblage and resolution of inflammatory responses (Markus et al. 2007, 2018). During disease-free conditions, increased MEL levels at night prevent leukocytes from crossing the endothelium (Markus et al. 2018). Otherwise, during inflammatory assemblage, innate immune responses inhibit central MEL secretion by the pineal gland contributing to a fast migration of leukocytes to the inflammatory site (Markus et al. 2018). Simultaneously, innate responses stimulate local MEL production by the immune-competent cells in damaged tissues, a process regulated by NF-κB (da Silveira Cruz-Machado et al. 2012, 2017). This switching in the MEL source from the pineal gland to immune-competent cells in response to inflammatory stimuli is called the immune-pineal axis (Markus et al. 2007, 2013, 2018). It is worth highlighting the interaction between the immune-pineal and the HPA/I axis, in which glucocorticoids have dual effects on pineal MEL synthesis. During the assemblage of an inflammatory response, HPA/I axis activation contributes to blocking MEL synthesis; in contrast, in the recovery phase, HPA/I axis activation contributes to the restoration of pineal MEL synthesis (Fernandes et al. 2017; Markus et al. 2018). In general, this effect is mediated by the intensity of the stress response, with high glucocorticoid levels reducing MEL synthesis, while lower levels result in its potentiation (Markus et al. 2018).

Nearly all the knowledge about the inflammatory process has been obtained from studies with endothermic model organisms (e.g., mostly small mammals and birds; Medzhitov 2008; Chrousos 2010; Naidu et al. 2010; Ashley et al. 2012; Evans et al. 2015). Still, little is known about the immune response in ectotherms (Zimmerman et al. 2010, 2014, 2017), especially inflammation in amphibians (Robert and Ohta 2009; Xi et al. 2017; Gardner et al. 2018). A major reason is that although cytokines’ biological activity is highly conserved, cross-reactivity of antibodies between species is limited due to subtle differences in protein sequences (Scapigliati et al. 2006). For amphibians, there are several cell markers and monoclonal antibodies available for use with the model system *Xenopus* (Robert and Ohta 2009), with no reports if those reagents cross-react with other amphibians species. Therefore, reagent development is critical to extending this research line (Zimmerman et al. 2010). In that regard, Gardner et al. (2018) used the transdermal corticosterone (CORT) treatment and lipopolysaccharide (LPS) stimulation to obtain a transcriptome of spleen tissue from a population of cane toads (*Rhinella marina*), a worldwide invasive species. The authors reported upregulated genes coding for cytokines involved in typical innate responses such as phagocytic cell recruitment, inflammation, and lymphocyte differentiation in response to LPS, while toads receiving the CORT transdermal application in addition to LPS injection showed downregulation of genes involved with cell-mediated immunity (Gardner et al. 2018).

LPS is a component of the Gram-negative bacteria cell walls, commonly used to induce systemic inflammation (Beutler and Rietschel 2003; Lu et al. 2008). When LPS binds to TLR4, it triggers both increased expression of several proinflammatory cytokines (e.g., TNF-α, IL1-β, IL-6, IL-8; Alexander and Rietschel 2001; Lu et al. 2008; Ramachandran 2014) and the complement system activity (Ricklin et al. 2010; Merle et al. 2015). Additionally, LPS stimulates the HPA/I axis (Harbuz et al. 1999; Bornstein et al. 2006) and inhibits MEL synthesis (Tamura et al. 2010; Piesiewicz et al. 2012; Markus et al. 2018) in mammals and birds. While the basic components of innate and adaptive immune defenses in frogs are known, the impact of glucocorticoids and melatonin on the inflammatory assemblage in toads is not well studied. To further explore the inflammatory processes and fill some gaps in understanding how a pathogen can activate immune and endocrine mediators during the assemblage of the inflammatory process in wild-caught anurans, we submitted *Rhinella diptycha* native toads from Brazil to an LPS immune challenge. We hypothesized that an immune challenge would act as a physiological stressor, activating the HPA/I axis and the immune response and inhibiting central MEL secretion. The specific predictions were that 6 h post-injection, LPS-treated toads would have: (1) increased CORT levels, the main glucocorticoid in anurans, due to the HPA/I activation; (2) decreased MEL levels since inflammation and high glucocorticoid levels block pineal MEL synthesis; and (3) considering the time course of the inflammatory assemblage, we should detect mRNA expression upregulation of pro- (IL1-β and IL-6) and anti-inflammatory (IL-10) cytokines in the spleen.

## Materials and methods

### Species and collection site

*Rhinella diptycha* (formerly *Rhinella schneideri*; Lavilla and Brusquetti 2018) is a generalist toad species widely distributed in South America, including Cerrado, Atlantic Rainforest, and Northeast of Argentina (Maciel et al. 2010). Adult male toads (*N* = 16) were collected in the city of Botucatu (22°46′59.9″S, 48°28′28.1″O), Sao Paulo/Brazil, in November 2017. Toads were located by visual inspection, captured by hand, and placed in clear plastic bins (eight animals each) with lids with holes for air circulation. These animals were transported to the laboratory and kept individually in new plastic bins (20 L; 43.0 [L] × 28.5 [W] × 26.5 [H] cm) with lids that had holes for air circulation and free access to water within a controlled room in the Department of Physiology, Institute of Biosciences, University of Sao Paulo (23°33′45″S, 46°43′40″W). The temperature was set to 21 ± 2°C, and the photoperiod was 13 h of light and 11 h of darkness (LD 13:11). After 7 days in captivity, body mass (0.01 g) and the snout-vent length (SVL; 0.01 mm) were measured, and the body index was calculated (unstandardized residuals of a linear regression of body mass as a function of SVL; Schulte-Hostedde et al. 2005) and considered as a proxy of body condition. We randomly assigned toads to study groups following the collection of morphometric measures, as described below.

Animals were collected under license from Instituto Chico Mendes de Conservação da Biodiversidade (ICMBio, #29896-1). All procedures realized were approved by the Ethics Committee from the Institute of Biosciences, University of Sao Paulo (CEUA: #242/2016).

### Lipopolysaccharide (LPS) treatment

Animals were equally and randomly divided into two groups (*N* = 8 by group), according to Gardner et al. (2018): (1) LPS: received an intraperitoneal injection of LPS (*Escherichia coli* O127:B8, Sigma-Aldrich, L3129, Saint Louis, MO, USA) in the concentration of 2 mg/kg diluted in amphibian phosphate-buffered saline (APBS: NaCl: 8 g; KCl: 0.2 g; Na_2_PO_4_: 1.44 g; KH_2_PO_4_: 0.24 g, diluted in 1.3 L of distilled water, pH 7.4); and (2) saline: received an equivalent volume of APBS. The injections were administered 20 min after the lights turned off (7 pm). This time was chosen because toads are nocturnal animals. During the night, they are active, foraging, and searching for mates, increasing the likelihood of encountering pathogens.

### Blood and tissue collection

Six hours post-treatment (1 am), toads were bled (800 μL of blood) via cardiac puncture with 1 mL syringes and 26 G × 1/2″ needles that were previously heparinized. Blood samples received an ID, were kept on ice (<1 h), and centrifuged (604 *g* × 4 min, 23°C) to isolate the plasma. Plasma samples were stored in a −80°C freezer and used to measure CORT and MEL levels. Toads were then euthanized by immersion in a lethal solution of benzocaine (0.2%). After death confirmation, spleens were collected, frozen in liquid nitrogen, and then transferred to a −80°C freezer for gene expression of cytokines involved in the inflammatory process (see the section “Cytokines’ selection”).

### Hormonal assays

This assay was run following the methods in Assis et al. (2017). In brief, 10 µL of each plasma sample was transferred to a glass test tube. Then, a volume of 3 mL of ether was added to each sample. Tubes were agitated for 30 s and centrifuged (583 *g*, 9 min, 4°C). Samples were taken to a −80°C freezer for 7 min and then the contents were transferred to another tube. Tubes were covered with a paper towel and kept in a laminar flow hood at room temperature (23 ± 2°C) until the evaporation of all ether (∼24 h). On the assay day, samples were resuspended in ELISA buffer, and CORT was assayed using ELISA kits (CORT number 500655; Cayman Chemical, Ann Harbor, MI, USA), according to the manufacturer's instructions. Intra- and inter-assay variations were 2.57% and 3.49%, respectively. The sensitivity of the assays was 23.11 pg/mL.

For MEL levels, 250 µL of plasma were extracted using silica columns (Waters Sep-Pak^®^ Vac, supplied in the IBL kit) and methanol. Samples were resuspended in ultrapure water, and MEL concentrations were determined through a commercial ELISA kit (IBL, RE54021, Männedorf, Switzerland), according to the manufacturer's instructions and previous studies conducted with amphibians (Barsotti et al. 2017; Titon et al. 2021a). The intra-assay coefficient of variation was 3%. The sensitivity of the MEL assay was 1.3 pg/mL.

### Molecular data

#### Cytokines’ selection

We selected the following cytokines: the proinflammatory TNF-α, IL-1β, IL-6, developed by Gardner et al. (2018); the anti-inflammatory IL-10, the proinflammatory IFN-γ, and the complement protein C1s, developed by our research group in Brazil, using *R. marina* transcriptome provided by Gardner et al. (2018); and the housekeeping gene β-actin developed by Halliday et al. (2008). All primers were purchased from Thermo Fisher Scientific (Waltham, MA, USA) (Table 1).

**Table 1:** Sequences of primers used for gene expression of the target proteins

Primers	Sequence (5′ → 3′)	Animal model	Reference
β-Actin (117 bp)	**F:** ATGACACAGATAATGTTTGAGAC	*Rhinella marina*	Halliday et al. (2008)
	**R:** ATCACCAGAGTCCATCACAAT		
TNF-α (109 bp)	**F:** ACCAACGCCTTCAAAGATGG	*Rhinella marina*	Gardner et al. (2018)
	**R:** ATCTTTGCCCAGTGAACACC		
IL-1β (110 bp)	**F:** GAGAACATTGCGCAAGAAGC	*Rhinella marina*	Gardner et al. (2018)
	**R:** AAATAGAGTTGACGGCCTGC		
IL-6 (112 bp)	**F:** CAGTGATCTCCTGACGTTCC	*Rhinella marina*	Gardner et al. (2018)
	**R:** AGCATTTGCCAAGGAGATGG		
IL-10 (140 bp)	**F:** AGGACAAGCTCCTAGACCTGA	*Rhinella marina*	Developed
	**R:** TCCAACTGCCTTGTACATCCC		
IFN-γ (84 bp)	**F:** TGTGAGCAGCCACAAGACAT		
	**R:** GCATGCGGCCTTGGATCTTA	*Rhinella marina*	Developed
C1s (102 bp)	**F:** GCTGCCTGTACGACAGTCTT	*Rhinella marina*	Developed
	**R:** CTTCATTGCTGCCCGATTCG		

#### RNA extraction

Spleens (∼50 μg) were transferred to 1.5-mL sterile microtubes and homogenized in 750 μL cold (4°C) Trizol reagent (Thermo Fisher Scientific, 15596018). Then, microtubes were placed under low agitation in an orbital shaker for 30 min at room temperature (23°C). The next step was adding 200 μL cold chloroform (4°C), followed by homogenization (5-time slowly inversion with hands), agitation in the orbital shaker for 10 min (low agitation), and centrifugation (12,000 *g*, 15 min, 4°C). The clear and watery layer (containing the RNA and DNA) was transferred to a new microtube. In these new microtubes, 500 μL cold (−20°C) isopropyl alcohol was added to precipitate RNA. Samples were agitated (10 s) and incubated (30 min, −20°C freezer), followed by centrifugation (12,000 *g*, 15 min, 4°C). The supernatant was disposed of, and 1 mL cold ethanol (75%, −20°C) was added to the microtubes to wash and remove impurities. The samples were centrifuged again (12,000 *g*, 15 min, 4°C), the supernatant disposed of, and the remaining ethanol was allowed to evaporate. The dried pellets were resuspended with RNAse-free water, and the concentration and quality of samples were measured in a Nanodrop spectrophotometer at A260/A280 (Nanodrop ND1000, Thermo Fisher Scientific).

For genomic DNA elimination, a solution was prepared using 4 μg RNA, 2 μL DNAse I (Thermo Fisher Scientific, EN0521), 2 μL Buffer 10× (10% of the total volume of the solution), and water to fulfill a total volume of 20 μL. Samples were incubated (37°C, 60 min), and 2 μL EDTA (25 mM) was added to a new cycle: 65°C, 10 min, followed by 8°C withholding (My Genie 96 Thermal Block, Bioneer Corporation, South Korea).

#### Conversion of RNA into complementary DNA (cDNA)

The reverse transcription was performed using 2 μg purified RNA, 2 μL random primer (100 μM, Thermo Fisher Scientific, SO142), 2 μL dNTPs (10 mM, Thermo Fisher Scientific, R0192), and 2 μL reverse transcriptase enzyme (200 U RevertAid H minus Reverse Transcriptase kit, Thermo Fisher Scientific, EP0451) in RNAse-free water to obtain a total volume of 40 μL, following the manufacturer’s instructions. Samples were placed in a thermocycler, following this program: 25°C for 10 min; 42°C for 120 min; 70°C for 10 min and withholding at 4°C.

#### Primers test

Using the polymerase chain reaction (PCR) method, each sample was prepared with: 7.5 μL 2× DreamTaq Master Mix (Thermo Fisher Scientific, K1081), 0.15 μL target primer (10 μM; 0.075 μL forward primer + 0.075 μL reverse primer), 50 ng cDNA, and water to complete the final volume of 15 μL. The reaction was performed in thermocycler according to the following steps: 1 cycle at 95°C for 5 min, 40 cycles of 95°C for 1 min, 60°C for 30 s and 72°C for 15 s, 1 cycle of 72°C for 5 min and withholding at 8°C. All PCR products were analyzed by electrophoresis (2% agarose gel). Excluding the TNF-α, all the other targets (IL-1β, IL-6, IL-10, IFN-γ, and C1s) were amplified, with fragments having the right base pair length. The absence of amplification of TNF-α suggests that even phylogenetically close species may not always show cross-reactivity. A new purified solution of the PCR product was made for the quantitative PCR (qPCR) efficiency test. To verify the specificity of the primers, cold (4°C) sodium acetate (10% of the PCR product total volume) was added in each sample. The microtubes were agitated, and we added twice the volume of cold ethanol (100%, 4°C). The microtubes were agitated and transferred to a −20°C freezer for 15 min, and then centrifuged (12,000 *g*, 4°C, 30 min). The supernatant was disposed of, and 1 mL cold ethanol (70%, 4°C) was added to the microtubes and centrifuged (12,000 *g*, 4°C, 10 min). The supernatant was disposed of, and the remaining ethanol could evaporate. Pellets were resuspended in 30 μL of TE (Tris 10 mM + EDTA 1 mM; Tris-HCl: 1.576 g; EDTA: 292 mg [pH 8.0], to a final volume of 100 mL of RNAse-free water) and quantified by a Nanodrop spectrophotometer. The calculations were based on fragment length and molecular mass to determine the copy numbers of each PCR product.

The purified samples were serially diluted (10-fold) for each primer, and a qPCR was performed using a thermocycler (Applied Biosystems StepOne^™^ Real-Time PCR System, Thermo Fisher Scientific) and following this program: 95°C for 10 min, followed by 40 cycles of 95°C for 15 s and 60°C for 1 min. Cycles were followed by a melting curve of 95°C and a gradient of 60–95°C, with increments of 1°C. At the end of the test, results were obtained using StepOne Software Version 2.3. All primers showed specificity (*R*^2^ ≥ 0.96 and efficiency ≥88%; Fig. S1).

#### RT-qPCR

For RT-qPCR, we performed a reaction mix containing 10 μL SYBR Green 2× (Thermo Fisher Scientific, K0223); 0.1 μL target primer (10 μM; forward + reverse mix); 5 μL sample (cDNA 50 ng), and water to obtain a final volume of 20 μL. Reactions were made following this program: 95°C for 10 min, followed by 40 cycles of 95°C for 15 s and 60°C for 1 min in a thermocycler (Applied Biosystems StepOne^™^ Real-Time PCR System). The cycles were followed by a melting curve of the gradient of 60–95°C, with increments of 1°C. At the end of the test, results were obtained using StepOne Software Version 2.3.

### Statistical analysis

Endocrine (CORT and MEL), molecular (IL-1β, IL-6, IL-10, IFN-γ, and C1s), and morphometric (body mass and SVL) data were initially evaluated with a Shapiro–Wilk normality test. Some variables showed an absence of normality (MEL, IL-10, C1s, and body mass) and were subjected to the nonparametric independent samples test of Mann–Whitney. The variables fitting the parametric assumptions (CORT, IL-1β, IL-6, IFN-γ, and SVL) were analyzed through independent samples *t*-test. Correlations were also used to investigate possible relations among the different variables inside each group. Gene expression rate was obtained by relative quantification using ∆∆CT method (Livak and Schmittgen 2001) and are shown as a fold change of each cytokine to the housekeeping gene β-actin. Any outliers identified from a *Z*‐score test were removed, and data were analyzed using the SPSS Version 26 for Windows.

## Results

**Fig. 1:**
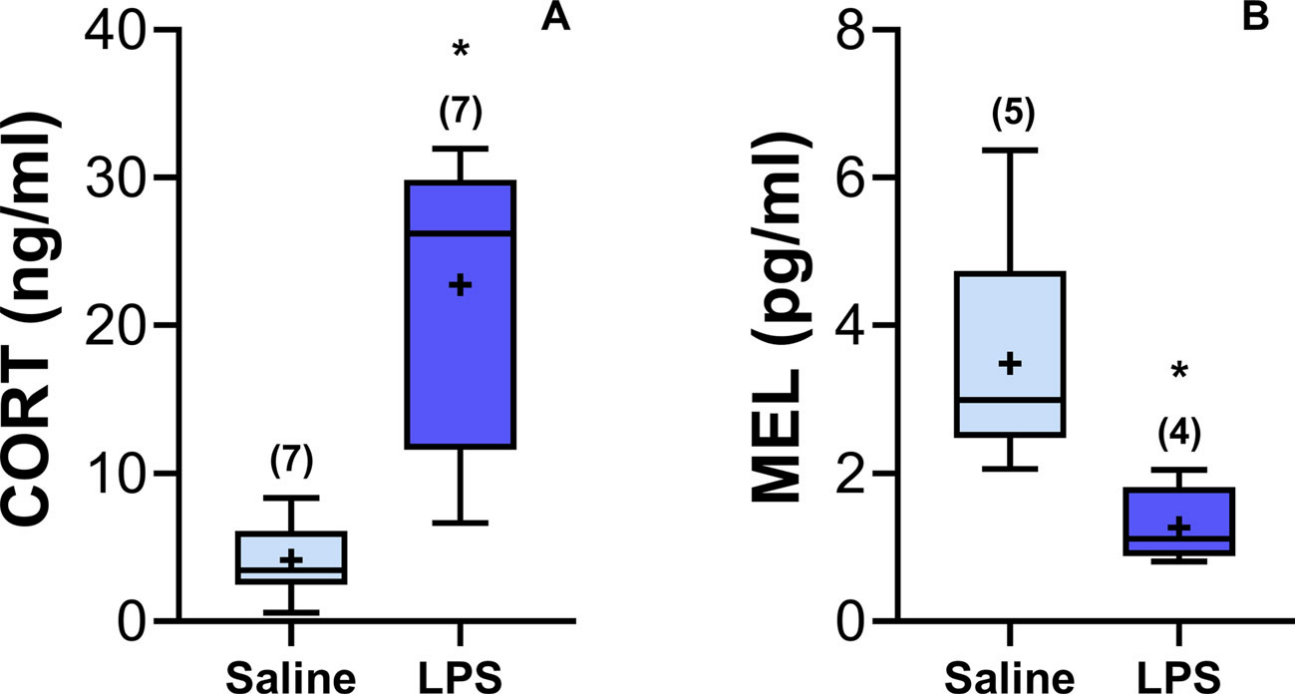
*Rhinella diptycha* endocrine mediators after immunological challenge with LPS. **(A)** Plasma corticosterone levels. **(B)** Plasma melatonin levels. The rectangle of the box plot represents interquartile range (IQR), in which upper and lower lines inside it represent first and third quartiles, respectively; central lines represent the median; and whiskers represent upper and lower limits of 1.5 times IQR. The symbol (+) represents the mean values, and the *N* is indicated in parentheses. Asterisks (*) denote significant differences (*P* ≤ 0.05) between the LPS and saline groups.

**Fig. 2:**
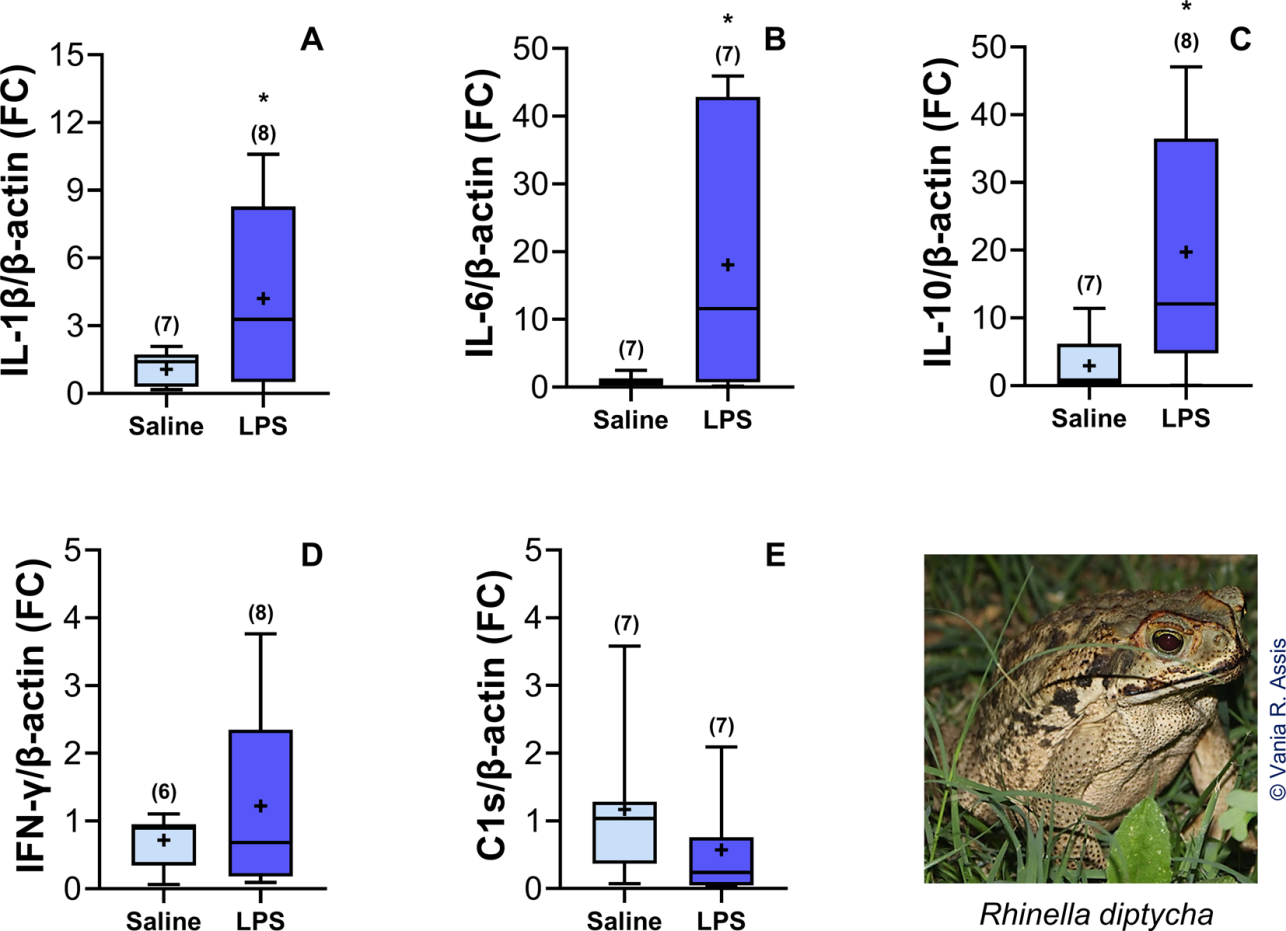
Gene expression in *Rhinella diptycha*, after immunological challenge with LPS. **(A–E)** Gene expression rate (fold change) of cytokines IL-1β, IL-6, IL-10, IFN-γ, and C1s, respectively. The rectangle of the box plot represents interquartile range (IQR), in which upper and lower lines inside it represent first and third quartiles, respectively; central lines represent the median; and whiskers represent upper and lower limits of 1.5 times IQR. The symbol (+) represents the mean values, and the *N* is indicated in parentheses. Asterisks (*) denote significant differences (*P* ≤ 0.05) between the LPS and saline groups.

Descriptive statistics of endocrine, molecular, and morphometric variables for *Rhinella diptycha* toads can be found in Table 2. LPS-treated toads showed five times higher CORT levels (Table 3, Fig. 1A) and three times lower MEL levels (Table 4, Fig. 1B) than the saline-treated ones. Concerning cytokine gene expression, we found upregulation of IL-1β, IL-6, and IL-10 in the LPS-treated group (Tables 3 and 4, Fig. 2A–C), contrasting with no effect on IFN-γ and C1s proteins (Tables 3 and 4, Fig. 2D and E). The proinflammatory cytokine IL-1β was upregulated by four-fold in the LPS-treated group compared with the saline-treated group, while the proinflammatory IL-6 was upregulated 20-fold, and the anti-inflammatory IL-10 by seven-fold. There was a positive correlation between IL-1β and IL-6 (*r* = 0.861; *P* = 0.013) inside the LPS group.

**Table 2:** Descriptive statistics of endocrine, molecular, and morphometric variables for *Rhinella diptycha* toads

Parameters	*N*	Minimum	Maximum	Mean ± SD
Saline				
CORT (ng/mL)	7	0.59	8.36	4.14 ± 2.55
MEL (pg/mL)	5	2.06	6.37	3.49 ± 1.66
IL-1β (FC)	7	0.17	2.08	1.08 ± 0.79
IL-6 (FC)	7	0.20	2.47	0.87 ± 0.82
IL-10 (FC)	6	0.04	11.40	2.93 ± 4.49
IFN-γ (FC)	6	0.07	1.11	0.72 ± 0.39
C1s (FC)	7	0.07	3.58	1.17 ± 1.15
Body mass (g)	8	70.52	238.06	143.51 ± 56.15
SVL (mm)	8	87.91	126.24	107.05 ± 13.46
LPS				
CORT (ng/mL)	7	6.65	31.95	22.77 ± 9.81
MEL (pg/mL)	4	0.81	2.05	1.27 ± 0.54
IL-1β (FC)	8	0.01	10.60	4.21 ± 4.06
IL-6 (FC)	7	0.16	45.94	18.07 ± 19.73
IL-10 (FC)	7	0.07	47.08	19.72 ± 18.56
IFN-γ (FC)	8	0.09	3.76	1.22 ± 1.33
C1s (FC)	7	0.03	2.10	0.57 ± 0.73
Body mass (g)	8	87.16	185.58	117.77 ± 30.16
SVL (mm)	8	95.21	113.50	101.32 ± 6.12

CORT: corticosterone plasma levels; MEL: melatonin plasma levels; SVL: snout-vent length.

**Table 3:** Comparison of endocrine, molecular, and morphometric variables between saline- and LPS-injected toads

Variables	MD	*t*	DF	*P* (1-tailed)
CORT (ng/mL)	−18.630	−4.861	6.8	**≤0.001**
IL-1β (FC)	−3.136	−2.138	7.6	**0.034**
IL-6 (FC)	−17.196	−2.303	6.0	**0.031**
IFN-γ (FC)	−0.503	−1.011	8.5	0.170
Body index	1.652	0.294	14	0.387
SVL (mm)	5.736	1.097	14	0.146

For parametric variables, a *t*-test was performed with endocrine (CORT), molecular (IL-1β, IL-6, IFN-γ), and morphometric (body index and SVL) parameters as dependent variables and treatment (saline or LPS) as a factor. DF: degrees of freedom; MD: mean difference (saline—LPS); FC: fold change; CORT: corticosterone plasma levels; SVL: snout-vent length. Variables with *P* significant at 0.05 are highlighted in bold.

**Table 4:** Comparison of endocrine, molecular, and morphometric variables between saline- and LPS-injected toads

Variables	MD	*Z*	*U*	*P* (1-tailed)
Body mass (g)	25.738	−0.945	23.0	0.191
IL-10 (FC)	−16.791	−2.074	6.5	**0.018**
C1s (FC)	0.600	−1.469	13.0	0.083
MEL (pg/mL)	2.216	−2.449	0.0	**0.008**

For non-parametric variables, a Mann–Whitney test was performed with endocrine (MEL), molecular (IL-10 and C1s), and morphometric (Body Mass) parameters as dependent variables and treatment (saline or LPS) as a factor. MD: mean difference (saline—LPS); FC: fold change; MEL: melatonin plasma levels. Variables with *P* significant at 0.05 are highlighted in bold.

## Discussion

Our results demonstrate that the amphibians have similar responses to mammals and birds in terms of endocrine mediators and immune function during an inflammatory response. Following an LPS stimulation, wild-caught toads increased CORT levels, decreased MEL levels, and upregulated splenic mRNA of pro- and anti-inflammatory cytokines. LPS-treated toads showing higher CORT levels than the saline-treated toads support our prediction of activation of the HPA/I axis due to LPS treatment (Coleman et al. 1993; Harbuz et al. 1999; Herman et al. 2013). In mammals, LPS binds to TLR4, which is essential for innate recognition of Gram-negative PAMPs and is ubiquitous on most monocytes, macrophages, and epithelial cell types, being also present in adrenal cells (Bornstein et al. 2004, 2006) and the hypothalamus (Bornstein et al. 2006; Herman et al. 2013). The activation of the TLR4 by the LPS can then promote an elevation in CORT levels (Bornstein et al. 2006; Cain and Cidlowski 2017), a result already observed in toads, varying from 2 to 20 h post-LPS injection (Gardner et al. 2018, 2020). The inflammatory cytokines IL-1β and IL-6 can also stimulate the HPA/I axis alone or in synergy with each other (Chrousos 1995). Besides, there is evidence suggesting that IL-6 plays a central role in the immune stimulation of the HPA/I axis (Tsigos and Chrousos 2002). Therefore, the HPA/I activation is possibly related to the immune response in toads through the activation of TLR pathways, as well as through IL-1β and IL-6 signaling, increasing CORT levels. More studies using molecular methods to measure TLR4 pathway activation and their implications during the inflammatory process may help to understand its relationship with CORT levels and immune response against pathogens in free-living amphibians.

Regarding MEL levels, the lower values in the LPS-treated toads also support our prediction. In mammals, MEL stops being centrally produced in the pineal gland and starts being produced locally at the inflammatory site by immune cells when individuals are undergoing an inflammatory process, a phenomenon called immune-pineal axis activation (Markus et al. 2007, 2018). The existence of an immune-pineal axis in amphibians is yet to be confirmed since there is no evidence of increased local MEL production by amphibian immune cells challenged by TLR ligands. However, decreased circulating MEL levels in our toads indicate inhibition of MEL production in the pineal gland during infections in anurans, as previously described in mammals (Markus et al. 2007, 2018). Further studies analyzing plasma MEL levels and the gene expression of enzymes related to the MEL production pathway, such as arylalkylamine *N*-acetyltransferase (AANAT) and acetylserotonin O-methyltransferase (ASMT), in the pineal gland and extracellular fluid of *in vitro* LPS-stimulated peritoneal leukocytes, should help to clarify this matter.

Concerning cytokine gene expression, the profile we found (IL-6 > IL-10 > IL-1β) is according to the timeline of events happening at an inflammatory site, already described for mammals and reptiles (Chrousos 1995; Dienz and Rincon 2009; Zimmerman et al. 2014). IL-1β and IL-6, the first proinflammatory cytokines secreted, stimulate their own production, and IL-1β also stimulates IL-6 production (Chrousos 1995; Dienz and Rincon 2009; Zimmerman et al. 2014). The positive correlation between IL-1β and IL-6 inside the LPS group indicates the functional interrelation between these cytokines in our toads. Interestingly, IL-6 inhibits the secretion of IL-1β while stimulating glucocorticoid secretion (Chrousos 1995; Hopkins 2003). The glucocorticoids, in turn, inhibit the production of both proinflammatory cytokines (IL-1β and IL-6), while stimulating the secretion of anti-inflammatory cytokines such as IL-10 and IL-4 (Chrousos 1995; Elenkov and Chrousos 2002). In studies with *Xenopus tropicalis*, frogs injected with LPS showed a 15-fold increase in IL-10 gene expression compared with the saline-treated frogs, 24 h post-injection (Qi et al. 2015). In this way, it is possible that this cytokine would have a greater expression in later hours, inducing a further decrease of IL-1β, IL-6, and other proinflammatory mediators, thus regulating the inflammatory response. Our results show that these dynamics, cytokines stimulating/inhibiting other cytokines, and stimulating endocrine mediators (CORT), which in turn modulate pro- and anti-inflammatory cytokines, are present in amphibians. However, exploring other time points are crucial to understand the time course of these relationships better.

Meanwhile, IFN-γ and C1s were not differentially expressed between LPS- and saline-injected toads. For IFN-γ, the absence of effects might be related to the time post-application since Qi and Nie (Qi and Nie 2008) reported increased IFN-γ 24 h post-LPS injection in frogs (*Xenopus tropicalis*). Also, we have higher CORT levels 6 h post-injection, and glucocorticoids suppress the transcription of IFN-γ (Webster et al. 2002; Franchimont 2004). Regarding C1s, tissue choice might have reduced the likelihood of detecting gene expression. Hepatocytes produce most of the complement system proteins in mammals and amphibians, although immune and endothelial cells also produce these proteins (Kunnath-Muglia et al. 1993; Zhou et al. 2016). In a study with the rock bream fish (*Oplegnathus fasciatus*), the authors found upregulation of this protease in the liver of individuals infected with bacteria, obtaining up to five-fold gene expression of C1s in the liver 6 h post-injection (Godahewa et al. 2015). Exploring the expression of cytokines and complement proteins in subsequent times post-LPS injection and different organs might help us better understand the time frame of their production and whether they are tissue specific in toads.

Together, the interconnection among the immune and endocrine mediators we found in *R. diptycha* toads matches the inflammatory response's progression, considering we collected the data 6 h post-LPS injection, including the absence of TNF-α, which might be found earlier. Indeed, experiments analyzing cytokines’ gene expression in cane toads 2 h post-injection found upregulated TNF-α, IL-1β, and IL-6 (Gardner et al. 2018) but not IL-10 gene expression. Moreover, the cytokine with higher gene expression being IL-6 agrees with the high CORT levels we found. Additionally, high plasma CORT levels and the production of proinflammatory cytokines are both associated with a reduction in pineal MEL synthesis.

## Conclusion

Our results confirm that, as in other vertebrates, toads facing an immunological challenge have increased cytokine expression related to the inflammatory process, such as IL-1β, IL-6, and IL-10. In addition, the fact that IL-6 and IL-10 had higher gene expression than IL-1β 6 h after the immunological challenge with LPS suggests that there may have been a shift from the alarm to the resolution phase of the inflammatory process within our study time frame. We suggest that the increased plasma CORT levels may be associated with the HPA/I axis activation directly by LPS or indirectly by proinflammatory cytokines. Furthermore, the reduction in MEL plasma levels in LPS-injected toads compared with saline-injected toads suggests the presence of the immune-pineal axis in amphibians, an exciting possibility that warrants further investigation.

In terms of the cytokines, it is possible that shortly after the LPS injection, we could detect TNF-α gene expression and higher values for IL-1β, culminating with the increase in the IL-6 production we found 6 h post-injection. IL-6 would then regulate the inflammatory response, stimulating the CORT production and release, decreasing MEL synthesis, and initiating the anti-inflammatory process, ending up with the action of the anti-inflammatory cytokine IL-10. Future studies, including different times post-injection and other tissues (e.g., liver), might help better understand the dynamics of the endocrine and molecular variables, considering the beginning, progression, and resolution of the inflammatory process in *R. diptycha* toads.

## Supplementary Material

obab025_Supplemental_File

## Data Availability

The original data used in this manuscript is available at the Mendeley Data, through the DOI: http://dx.doi.org/10.17632/r84m23jrzj.1.
