# Supplementary material for: Lipopolysaccharide Regulates Pro- and Anti-Inflammatory Cytokines, Corticosterone, and Melatonin in Toads
Source: Integr Org Biol. 2021 Aug 28;3(1):obab025. doi: 10.1093/iob/obab025 (PMC8475549; doi:10.1093/iob/obab025)
Supplement: obab025_Supplemental_File [file obab025_supplemental_file.pdf]

Supplementary Materials for

LPS regulates pro and anti-inflammatory cytokines, corticosterone, and melatonin in toads

This file includes:

Figure S1

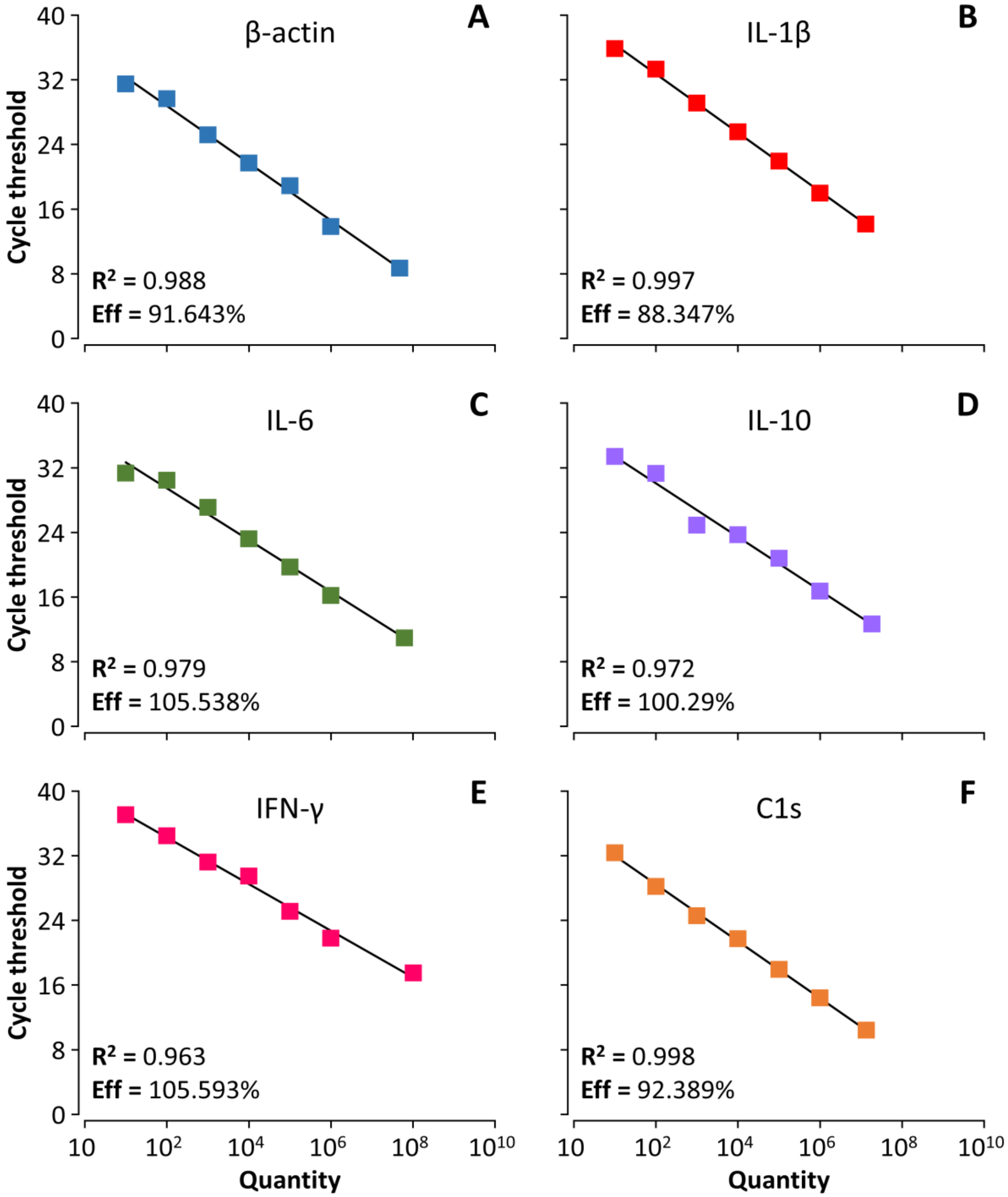

Figure S1. Primers efficiency curves confirming the amplification of fragments of interest (cytokines) in the spleen of *Rhinella diptycha* toads 6h post-LPS-injection. **A)** β-actin. **B)** Interleukin-1β. **C)** Interleukin-6. **D)** Interleukin-10. **E)** Interferon-γ. **F)** Complement component 1s. **R^2:** Coefficient of determination; **Eff:** efficiency percentage of the PCR amplification.
